# Aminoazo dye-protein-adduct enhances inhibitory effect on digestibility and damages to Gastro-Duodenal-Hepatic axis

**DOI:** 10.1371/journal.pone.0170555

**Published:** 2017-04-21

**Authors:** Li-Yun Lin, Chiung-Chi Peng, Yeh Chen, Boa-Chan Huang, Chun Chao Chang, Robert Y. Peng

**Affiliations:** 1 Department of Food And Applied Technology, Hungkuang University, Shalu County, Taichung City,Taiwan; 2 Graduate Institute of Clinical Medicine, College of Medicine, Taipei Medical University, Taipei, Taiwan; 3 Research Institute of Biotechnology, School of Medicine and Nursing, Hungkuang University, Sec. 6, Taiwan Boulevard, Shalu District, Taichung City, Taiwan; 4 Division of Gastroenterology and Hepatology, Department of Internal Medicine, Taipei Medical University Hospital, Taipei, Taiwan; 5 Division of Gastroenterology and Hepatology, Department of Internal Medicine, School of Medicine, College of Medicine, Taipei Medical University, Taipei, Taiwan; 6 Graduate Institute of Medical Sciences, Taipei Medical University, Xin-Yi District, Taipei, Taiwan; Virginia Commonwealth University, UNITED STATES

## Abstract

4-Dimethylaminoazobenzene (*DAB*, methyl yellow, or butter yellow), a human carcinogen, has been banned for use in foods since 1988. In 2014, *DAB* adulteration in Tofu occurred in Taiwan. We hypothesize that *DAB* can form [*DAB*•*SBP*]_adduct_ adduct with soybean protein (SBP) which could damage Gastro-Duodenal-Hepatic axis. Sprague-Dawley rats gavage fed [*DAB*•*SBP*]_adduct_ adduct revealed severely reduced body weight and damaged duodenum, liver, hepatic mitochondria, and spleen. Hepatic levels of glutathione and ATP were severely reduced. Serum GOT and GPT were substantially elevated. Analysis by the adsorption isotherm clearly revealed *DAB* formed very stable [*DAB*•*SBP*]_adduct_ adduct at 1:1 molar ration (Phase A). The equilibrium constant of this colloidal adduct [*DAB*•*SBP*]_adduct_ was *K*_*eqA*_ = ∝, behaving as the most stable and toxic species. At higher protein concentration (Phase C) it formed conjugate [*DAB*×*SBP*_gross_]_conjugate_, with *K*_*eqC*_ = 3.23×10^−2^ mg/mL, implicating a moderately strong adsorption. The *in vitro* pepsin digestibility test showed apparently reduced digestibility by 27% (by Ninhydrin assay) or 8% (by Bradford assay). Conclusively, this is the first report indicating that [*DAB*•*SBP*]_adduct_ potentially is capable to damage the Gastro-Duodenal-Hepatic axis.

## Introduction

Azo dyes are compounds characterized by their vivid colors and provide excellent coloring properties. Azo dyes account for approximately 60–70% of all dyes used in food, textile and leather industries[1]. 4-Dimethylaminoazobenzene (*DAB*, methyl yellow, or butter yellow), and o-amino-azotoluene are typical aminoazo dyes. Genetic toxicity of azo dyes has been demonstrated when the azo dyes are reduced by the intestinal bacteria[2]. Due to the toxicity, carcinogenicity and potential mutagenicity of thus formed aromatic amines, the use of certain azo dyes as food, textile and leather colorants, and the exposure of consumers ingesting the colored foods or using the textile and leather colored with azo compounds causes a serious health concern[1].

Early in 1935, Sasaki and Yoshida[3] demonstrated that administration of o-aminoazotoluene provoked progressive hepatocyte hyperplasia resulting in the formation of adenomas that became malignant in later days depending on the administration history[3].

*DAB*, a fat-soluble industrial yellow dye formerly had been used as a food additive. In rats aminoazo dye (butter yellow) induced liver damage associated with an irreversible increase of serum immunoglobin levels. *DAB* induced hepatomegaly, hepatic and renal dysfunction, liver cirrhosis and cancer, bladder cancer or osteoclasia during embryogenesis in dogs, and aspermatogenesis in mice [4] contact dermatitis, cough, wheezing, dyspnea, bloody sputum, urinary frequency, haematuria, dysuria, possible occupational carcinogen. *DAB* is considered hazardous by the 2012 Occupational Safety and Health Administration (OSHA) Hazard Communication Standard (29 CFR 1910.1200) [5]. A diversity of mechanistic pathways has been uncovered for azo dyes. The carcinogenic activation of *DAB* was suggested to proceed *via* oxidative mono-demethylation accompanied by N-oxidation, yielding the 4-hydroxy derivative of *DAB*, leading to the formation of a nitrenium ion which eventually attacks the position-8 of guanosine on DNA molecules [6]. Structural abnormalities of liver microsomes from rats fed 3'methyl-4-dimethylaminoazobenzene (3'Me-DAB), have been demonstrated by Arcos and Arcos[7]. Porter and Bruni[8] implicated that administration of (3'Me-DAB) could destroy organization of the rough endoplasmic reticulum and aggregation of the smooth form Porter and Bruni[8]. 3'Me-DAB forms t-RNA- azo dye complex in liver of rats, which is considered as the major target for this azo carcinogen[9]. Nonetheless, literature has indicated that the carcinogenicity of azo dyes varies with the species tested [10].

Since 1988 DAB has been banned and listed by the International Agency for Research on Cancer as a possible human carcinogen [4]. In the year 2009, European countries including Belgium and France detected the banned food colouring in Delhaize and Intermarche products [11,12]. Strikingly, on December 17, 2014, The Food and Drug Administration of Taiwan (FDAT) released a list of 36 food products adulterated with *DAB* soybean emulsifier supplied by Chien-Hsin Enterprise (Taiwan). The levels of *DAB* in these products ranged from 2.1 to 63.6 parts per billion (ppb). In reality, this *DAB* scandal was first set off by Hong Kong’s Center for Food Safety who discovered the prohibited dye in prepackaged pepper-flavored hard Tofu curds manufactured by Te-Chang Food Co. (Taiwan).

Tacal and Özer[13] demonstrated the formation of dye–protein adducts when highly electrophilic cationic dyes are quantitatively trapped in biological fluids and that the primary *in vivo* effects (e.g. toxicity) of such dyes most likely arise from ligand-type effects on multiple protein targets [13]. Furthermore, Saeed et al. reported the digestibility of dye-protein adduct can be reduced depending on the adduct characteristics [14].

By then, no sooner several colleagues had showed severe vomiting and gastritis after having ingested the yellow dye-colored bamboo shoot jam (Yi-Lan brand, Taiwan) for 12 h, than the motivation that inspired us to start with this experimentation. It was hypothesized that the adulteration of DAB into Tofu *in vitro* could form dye–protein adducts which may retard the digestibility of protein and simultaneously damage the gastroenteric system when ingested per os and it was supposed that 2–3 week-chronic ingestion should be able to induce the same event in the animal model. To verify this, we conducted the *in vitro* pepsin digestibility study as well as the *in vivo* animal experiment using the DAB-soybean protein model.

## Materials and methods

### Chemical and reagents

Pepsin was purchased from Sigma–Aldrich. The proteolytic activity of pepsin was ≥ 400U/mg protein. Methyl yellow (*DAB*) (95.0%) was purchased from Sigma–Aldrich (St. Louis, MO, USA). GOT and GPT ELISA kits were provided by Novatein Bioscience (Woburn, MA. USA).

Preparation of reference *DAB* curve: methyl yellow (Sigma Aldrich, St. Louis. MO, USA) was accurately measured and dissolved in 2 mL 0.1N HCl to make the stock solution. PBS (pH 3.2) was used to make dilutions to 10, 20, 30, 40, 50, and 60 μg/mL, respectively. The solutions were filtered through 0.45 μm Micropore and the absorbance was read at 430 nm to establish the standard curve.

Preparation of *DAB* digestibility test solution: *DAB* (11.265 mg) was accurately weighed, dissolved in 2mL 0.1 N HCl, and made volume to 10 mL with PBS (pH 3.2).

Preparation of soybean protein (*SBP*) solutions: *SBP* (ranging from 10 to 1000 mg, as indicated) were accurately weighed and transferred into 10 mL centrifuge tubes. To each tube 5 mL of PBS (pH 7.2) was added and agitated to facilitate the dissolution.

### Preparation of DAB-SBP hydrocolloids

DAB (11.265 mg) was accurately measured and dissolved in 10 mL PBS (pH 6.5) to make a stock solution of *DAB* (1.1265 mg/mL). For preparation of *DAB-SBP* hydrocolloid feed, to 5 mL of *SBP* solutions (40 mg *SBP*/mL) 300 μL of experimental *DAB* solution was added, agitated at 25°C for 30 min and centrifuged for 3 h at 12,000×g. The supernatant was collected for use. For adsorption experiment, to 5 mL of *SBP* solutions at different concentrations (0 to 220 mg SBP/mL) 300 μL of experimental *DAB* solution (1.1265 mg/mL) was added, agitated at 25°C for 30 min and centrifuged for 3 h at 12,000×g, the supernatant was read at 430 nm.

### Animals

This proposal for animal experiment had been approved by the Ethic Committee For Care of Animals and Animal Experiment of Hungkuang University (The affidavit of approval of animal use protocol has been attached herewith) (Supplement S-1). Sprague Dawley rats, age 4 weeks and weighing 140-150g, were purchased from LASCO Corporation (Taiwan). These animals were admitted and acclimated for the first two weeks in the animal room with good ventilation, controlled at RH 70–75%, 22±2°C, and 12/12h day/night cycle. The access to the water and the regular chow was *ad libitum*.

### Animal treatment

To mimic the toxicity of DAB enroute Tofu-ingestion, a dose of DAB at 281.6 ppb was estimated for gavage-fed rats having body weight around 400 g, which approximately corresponded to 4.5 folds of the maximum level of DAB (63.6 ppb) ever adulterated in Tofu. In brief, at the beginning of week 3, the rats were divided into three groups, 8 in each, and 2 rats per cage. Group 1 serving the control group was gavage fed 100 μL PBS daily. Group 2 was gavage-fed 100 μL *DAB*-*SBP* hydrocolloid (*DAB* 1.1265 mg/mL≈ 281.6 ppb) once only at the beginning. And group 3 was gavage-fed same amount of *DAB*-*SBP* hydrocolloid daily for a period of 14 days. The animals were monitored by their appetite, running activity, response to light and dark, and body weight change during the experimental period. It seemed that there was no apparent adverse effects when treated with DAB. On day 14, the body weight of animal was taken, and then the animals were fasted 12 h before euthanized. After the animals were CO_2_-euthenized, the organs hearts, kidneys, livers and duodenum were excised and rinsed with cold PBS buffer several times. The adhering water drops were soaked off with the kitchen tissue. The organs were weighed and the ratio of organ to body weight was accessed.

### Pathological change by Hematoxylin-Eosin staining

Part of the organs was subject to Hematoxylin-Eosin staining for histopathological examination at the Dr. J.-W. Liaw’s Lab at the Department of Pathology, College of Veterans of Chung-Hsing University (Taichung, Taiwan).

### Transmittance electron micrograph

After the organs were excised, rinsed and the adhering water was soaked off, part of the organs were immediately immersed in 20% formalin. The samples were delivered to the Electron Microscope Division of Chung-Shing University (Taichung, Taiwan) to carry out the TEM observation.

### Hepatic glutathione analysis

Part of hepatic lobe from individual rats were analyzed for reduced and total glutathione. The method was based on the determination of thiols by high-performance liquid chromatography with fluorescence detection [15]. Briefly, one mg of liver was placed in a test tube with 1 ml of 5% trichloroacetic acid containing 5mM EDTA, homogenized, vortexed, and centrifuged. Next, 50 μL of homogenate was transferred to a test tube with 950 μL of 5 mM EDTA containing 0.1 M borax (pH 9.3). Then, 100 μL aliquots were placed in duplicate tubes for each liver sample. Fluorobenzofurazan-4-sulfonic acid ammonium salt was added for reduced glutathione and tri-*n*-butylphosphine in acetonitrile was added to the other tube for total glutathione. High performance liquid chromatography with fluorescence detection was used for determining total and reduced glutathione and compared against calibration standards that were processed with each assay.

### Measurement of liver ATP by spectrophotometry

Liver ATP concentration was assayed by spectrophotometry using an assay kit (Nanjing Jiancheng Bioengineering Institute, Nanjing, China). The absorbance at 636nm was recorded and the concentration of ATP was expressed as millimoles per gram tissue (mmol/g tissue) [16].

### GOT and GPT assays

GOT and GPT were assayed according to the protocol instructed by the manufacturer (Novatein Bioscience, Woburn, MA. USA).

### The adsorption isotherm of *DAB* onto *SBP*

The whole scope of adsorption isotherm of *DAB* onto *SBP* ranging from 40 to 200 mg/mL was conducted at 25°C. To each 5 mL of *SBP* solution, 300 μL of experimental *DAB* solution (1.1256 mg/mL) was added, agitated at 25°C for 30 min and centrifuged for 3 h at 12,000×g. The absorbance of the supernatant was measured at 430 nm. The adsorption isotherm at 25°C was established by plotting absorbance vs. *SBP* concentrations. The absorbance of the supernatant was measured at 430 nm (hydrocolloid absorbance, *A*_*hyd*_) against the experimental *DAB* solution serving as the reference (reference absorbance, *A*_*ref*_). The difference of absorbance (Δ*A* = *A*_*ref*_*—A*_*hyd*_) was plotted against the concentration of *SBP*.

### *In vitro* pepsin digestibility test with DAB-SBP adduct

In vitro pepsin digestibility test was conducted by following Malinauskyte et al.[17], Sarkar et al.[18], and Sánchez-Chiang et al.[19]. As stomach is the front line digestive organ, only the pepsin digestibility test was performed to explore the effect of DAB on the digestibility of the soybean protein. The composition of the simulated gastric fluid (SGF) was as follows: 2.0 g NaCl, 6.9 g pepsin (601 U/mg), 7 mL conc. HCl, diluted to 1 L and pH adjusted to 1.2 using 1.0 M HCl. SGF was prepared according to procedure of Sarkar et al.[18]. The proteolytic activity of SGF was 36.6±0.8 U/mL [measured according to Sánchez-Chiang et al.[19].

In brief, judging from the adsorption isotherm, *DAB* digestibility test solution was added and thoroughly agitated for 30 min. The mixture was centrifuged at 12000×g for 3 h. The supernatant was discarded and the sediment was dissolved in 1 mL 2% KOH with agitation. The pH was adjusted with 1 M HCl to 1.2. SGF (2 mL) was added and incubated at 37°C for 30, 60, 90, 120, and 150 min respectively. The digestion reaction was terminated immediately at the end point by addition of 100 μL 2% KOH. The solution was centrifuged at 6000×g for 20 min. An aliquot of 100 μL of the supernatant was measured and subjected to amino acid ninhydrin analysis and Bradford protein assay. A control test was similarly performed using the same amount of *SBP* without *DAB*.

### Statistical analysis

Data obtained in the same group were analyzed using the SPSS 10.0 statistical software (SPSS, Chicago, IL, USA) and expressed in mean±S.D. An analysis of variance (ANOVAs) and Tukey’s test were used to analyze the variance and identify the significant differences between paired means at *p* < 0.05.

## Results and discussions

### Body/Organ weight affected by DAB-SBP hydrocolloids

No apparent morphological and behavioral changes were found during the experimental period. However prominent body weight change was observed. The body weight of group 2 rats (*DAB*-treated for one day) revealed to be the heaviest, reaching a body weight of 444.3±6.0g (*p*<0.05). The group 3 (*DAB*-treated for 2 weeks) rats was the lightest (395.8±5.0g), compared with 427.7±7.0g of the group 1 (the control) (*p*<0.05). A maximum deviation of 13.2% in body weight was found. The long term *DAB*-treated rats exhibited the heaviest livers (16.2±0.7g) comparing to 13.8±0.8g and 12.5±0.6g for the group 1 (control) and group 2 (Table 1). Long term *DAB*-treated group also characteristically revealed the heaviest kidneys and the lightest heart, however, the difference was insignificant. Nonetheless, the group 3 (*DAB*-treated for 2 weeks) rats exhibited the largest ratio of liver to body weight (0.041) and kidney to body weight (0.0088) (*p*<0.05), implicating *DAB* could have damaged these two organs (Table 1). In contrast, the heart weight and the ratio of heart to body weight did not show any significant changes (Table 1).

**Table 1:** Effect of methyl yellow on the body- and organ weight of SD rats.*

Group	Weight (g)				Ratio		
	BW	Liver	Kidney	Heart	LW/BW	KW/BW	HW/BW
Group 1	427.7±7.0^b^	13.8±0.8^b^	2.8±0.6	1.60±0.4	0.032	0.0065	0.0037
Group 2	444.3±6.0^a^	12.5±0.6^b^	2.9±0.5	1.60±0.3	0.028	0.0065	0.0038
Group 3	395.8±5.0^c^	16.2±0.7^a^	3.5±0.5	1.50±0.2	0.041	0.0088	0.0038

*Group 1 (the control): fed 100 μL PBS only per day. Group 2: administered *DAB*-*SBP* hydrocolloids (*DAB* 1.1265 mg/mL≈ 281.6 ppb) once only. Group 3: gavage-fed 100 μL *DAB-SBP* hydrocolloids (*DAB* 1.1265 mg/mL≈ 281.6 ppb) per day for a period of 14 days. Data from triplicate measurements were statistically treated and expressed in Mean±SD. Different superscripts in lower case indicate significantly different from each other (*p*<0.05).

Evidence has accumulated since the initial observations of Miller and Miller [20] suggesting that covalent bonding of aminoazo dyes to rat liver protein is essential for this group of carcinogens to manifest their activity [20]. Suggestively, DAB at the very early stage of feeding tended to form various conjugates with different hepatic enzymes, resulting in reduced body weight and damaged organs.

### *DAB*-protein adduct severely damaged the duodenum, but only moderately injured livers

**Fig 1:**
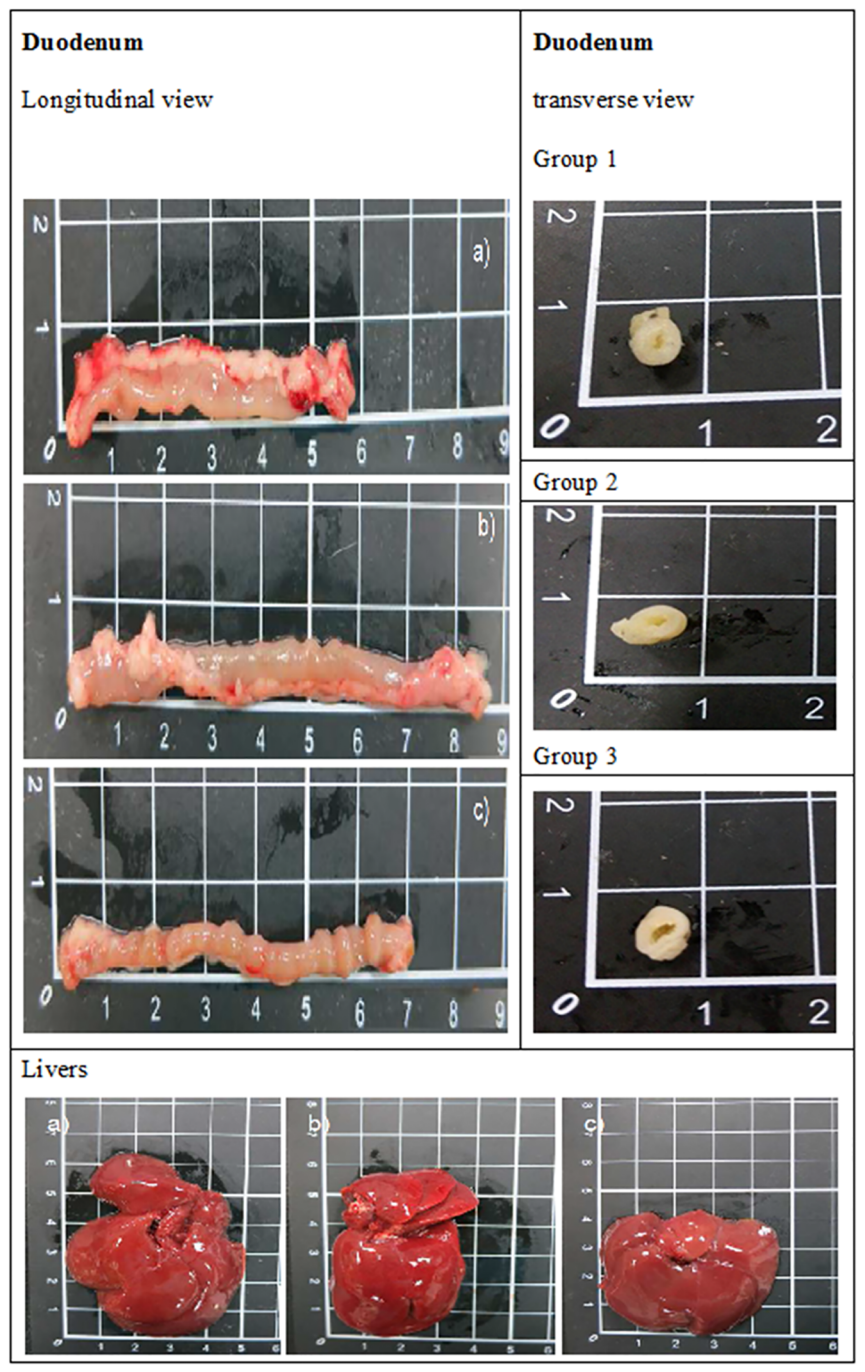
Duodenum and livers of Sprague-Dawley rats affected by methyl yellow. Methyl yellow was gavage-fed. Left column: longitudinal view of duodenum. Right column: transverse view of duodenum. a) Group 1, control. b) Group 2, treated with *DAB* for one day. c) treated with *DAB* for two weeks.

Normal duodenum appeared denser and exhibited brilliant transparency (Fig 1). The one-day *DAB*-treated duodenum seemed normal or only slightly damaged (Fig 1). As contrast, the duodenum of rats treated with *DAB* for two weeks was apparently smaller in circumference with pale outer look. The lumen of duodenum was totally destroyed leaving a larger cavity (Fig 1).

In contrast, although the gross appearance of livers of experimental rats seemed to be all normal (Fig 1), the liver weight differed greatly, exhibiting 13.8±0.8g, 12.5±0.6g, and 16.2±0.7g (*p*<0.05), respectively for the control, group 1 and group 2, respectively (Table 1). The ratio of liver to body weight also varied from 0.032, 0.028 to 0.041 (*p*<0.05), respectively (Table 1).

### Hematoxylin-Eosin staining revealed injured duodenum and liver tissues

**Fig 2:**
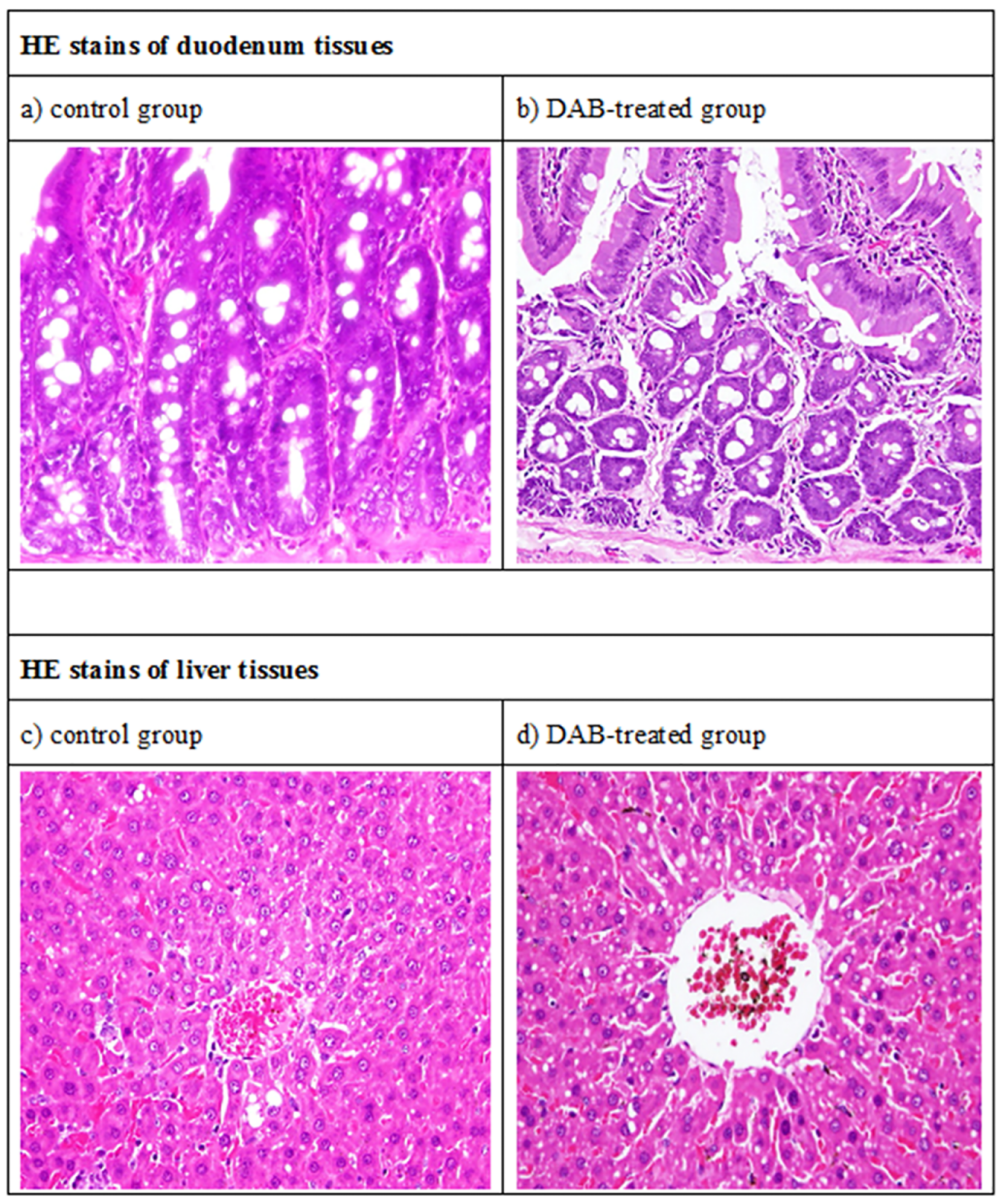
Hematoxylin-Eosin staining of duodenum and liver tissues. a) Duodenum control group. Normal elongated villi are seen. The villous to crypt ratio is at least 3:1. b) Duodenum of *DAB*-treated rats. Blunted and discrete villi, atrophy of central lacteal, rupture of columnar epithelium and much of oligomucous cells full of viscous fluid are noted, implicating the mitotic capability was disappearing and the cells were transforming into goblet cells (H&E, 400×). c) Liver of control group. Bright field image of an H&E stained section showing normal liver architecture and components of basic liver lobules with portal area and central venule. d) Livers of *DAB*-treated group. *DAB* enlarged and congested central venules and caused a bundle of swollen sinusoids and enlarged interhepatic plate spaces and endothelial cells are distinctly noted. The cavities fully filled up with plasma after damaged implicated a state of mild inflammatory (H&E, 400×).

The duodenum of control group with normal elongated villi can be clearly observed. The villous to crypt ratio was around ≥ 3:1, which is the regular size of normal duodenum (Fig 1). As contrast, the duodenum of *DAB*-administered rats were noted with blunted and discrete villi, atrophy of central lacteal, rupture of columnar epithelium and much of oligomucous cells full of viscous fluid, and some of the villi were affiliated with pathological increase of intraepithelial lymphocytes two weeks after *DAB* treatment, implicating that the mitotic capability was disappearing and the cells were transforming into goblet cells (Fig 2B) (H&E, 400×). Previously, similar finding had been reported by Orr et al.[21].

Histopathological examination by HE stain indicated that the livers of control group were all normal (Fig 2C). *DAB* enlarged and congested central venules and caused a bundle of swollen sinusoids and the interhepatic plate spaces. The cavities fully filled up with plasma after damaged by *DAB* implicated a state of mild inflammatory (Fig 2D).

Literature previously in detail cited by Opie[22] demonstrated similar pathogenesis in animals having received DAB, the earliest changes suggested cirrhosis occurring about hepatic veins and their smallest branches. Disintegration and disappearance of liver cell columns is associated with the accumulation of mononuclear cells [22].

### TEM micrograph

**Fig 3:**
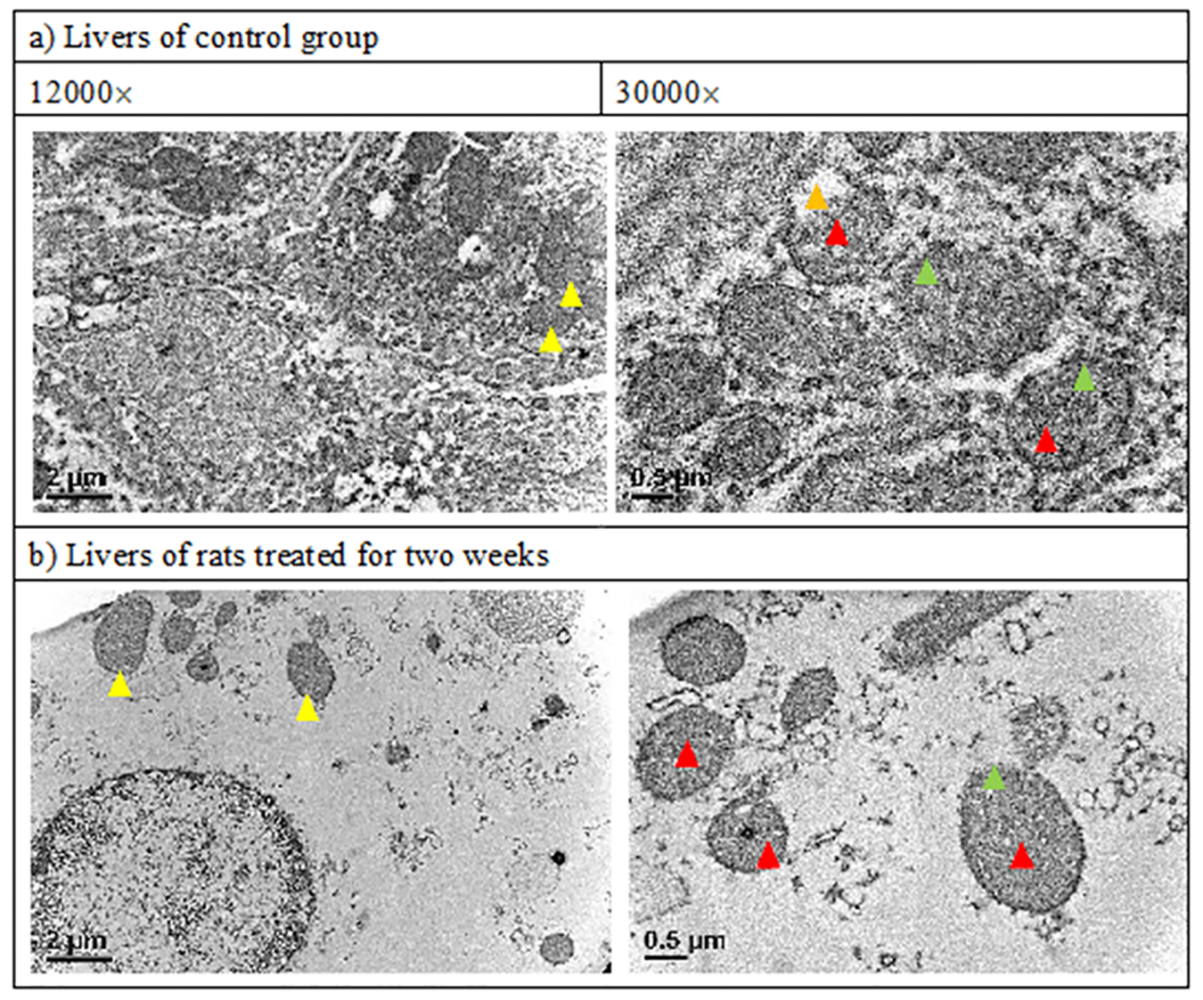
Transmittance Electron Micrograph of mitochondria. a) control. b) DAB-treated for two weeks.

The TEM image showed *DAB* after administered for two weeks severely damaged the mitochondria. As seen, the inner membrane structure, in particular, the cristae were all apparently destroyed (Fig 3).

### Hepatic glutathione level

Hepatic level of reduced glutathione (GSH) was severely reduced due by *DAB* administration for two weeks (Table 2). The level of GSH was reduced from 5.30±0.80 to 1.65±0.61 μmol/g (*p*<0.01). Substantial reduction of GSSG was also observed (*p*<0.05). On the other hands, levels of GOT and GPT were highly upregulated, reaching 188±26 and 56±14 μmole/μL (*p*<0.01), respectively comparing to the control (Table 2). Glutathione has multiple functions ranging from anti-oxidant defense to modulation of a diverse immune function and many physiological and biochemical conditions are related to low glutathione levels [23,24].

**Table 2:** Levels of GOT GPT, ATP and glutathione affected by administered methyl yellow–soybean protein adduct.*

	GSH, μmol/g	GSSG, μmol/g	GOT, μmol/μL	GPT, μmol/μL	ATP, μmol/g
Group 1	5.30±0.80^a^	0.06±0.02^a^	75±15^b^	25±11^b^	2.85±0.40^a^
Group 2	5.00±0.65^a^	0.07±0.03^a^	79±16^b^	28±13^b^	1.18±0.42^b^
Group 3	1.65±0.61^b^	0.04±0.02^b^	188±26^a^	56±14^a^	0.41±0.22^c^

*Group 1 (the control): fed 100 μL PBS only per day. Group 2: gavage-fed 100 μL *DAB*-*SBP* hydrocolloids (*DAB* 1.1265 mg/mL≈ 281.6 ppb) once only. Group 3: gavage-administered 100 μL *DAB*-*SBP* hydrocolloids (*DAB* 1.1265 mg/mL≈ 281.6 ppb) per day for a period of 14 days. Data from triplicate measurements were statistically treated and expressed in Mean±SD. Different superscripts in lower case indicate significantly different from each other (*p*<0.05).

### Hepatic ATP level

ATP was severely reduced due to *DAB* administration, correspondingly the ATP level was reduced from 2.85±0.40 to 0.41±0.22 μmol/g (*p*<0.01) (Table 2), compared to the cited normal (fed) value 3.21±0.2 μmol/g [25].

From the results shown in Fig 3 and Table 2, it is evident that mitochondrial structure was severely damaged by ingesting DAB for two weeks, in particular, the inner membrane cristae as well as the intermembrane space (lower panel in Fig 3B, 30,000×). The structure of inner membrane is highly complex, including all of the complexes of the electron transport system for oxidative phosphorylation, the ATP synthetase complex and transport proteins, and is freely permeable only to oxygen, carbon dioxide, and water [26]. As found, the intact structure of outer membrane totally disappeared when treated with DAB (Fig 3B, lower panel), suggesting that DAB could have completely destroyed both the transport and oxidative phosphorylation systems. Complex IV contains 13 different subunits encoded by both mitochondrial DNA and nuclear DNA. It receives an electron from each of the four cytochrome c molecules, and transfers it to one oxygen molecule, converting it into two molecules of water. In this process, it also binds to four proton molecules and translocates them across the membrane to establish electrochemical gradient, which is utilized for the synthesis of ATP [27].

### Hepatic GOT GPT levels

On the other hands, levels of GOT and GPT were highly upregulated after treating with DAB, reaching 188±26 and 56±14 μmole/μL (*p*<0.01) comparing to the controls 75±15 and 25±11 μmole/μL, respectively (Table 2). Obviously these levels may be higher for humans, but it corresponds well to data for rats [28].

### [*DAB*×*SBP*_*gross*_]_*conjugate*_ severely reduced pepsin digestibility

**Fig 4:**
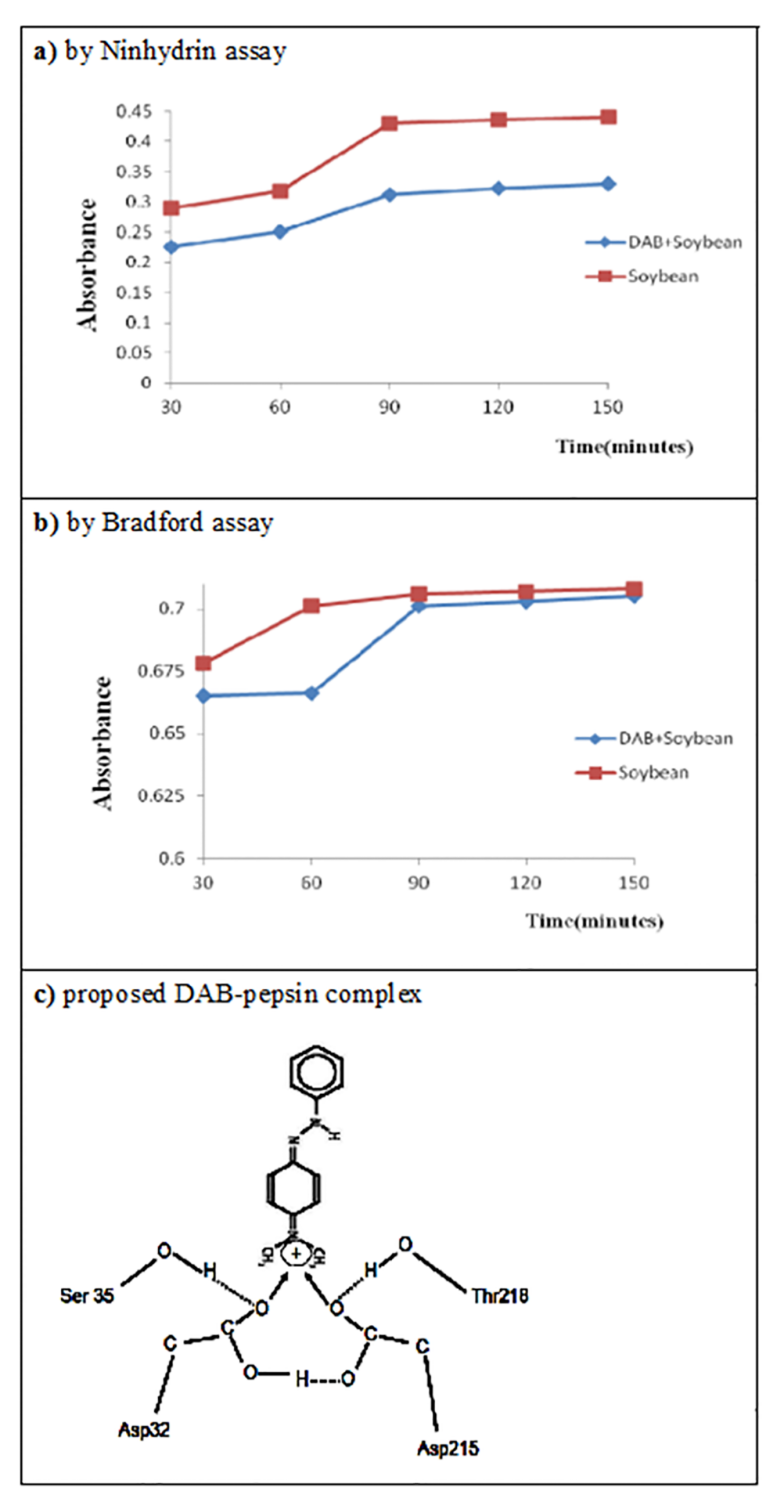
The *in vitro* pepsin digestibility test on *SBP* and [*DAB* × *SBP*_*gross*_]_*conjugate*_. a) by ninhydrin assay. b) by Bradford assay. c) schematic representation indicating the formation of DAB-Pepsin complex at the active sites of pepsin, Asp32 and Asp215. Ser35 and Thr218 help DAB trapping reinforcing the stability of DAB- pepsin complex.

The pepsin digestibility on soybean protein was substantially suppressed by the adduct [*DAB*•*SBP*]_*adduct*_ and the *DAB*-*SBP* adsorption conjugate, [*DAB*×*SBP*_gross_]_conjugate_. As seen, the ninhydrin assay revealed apparently the suppressed amino acid release from SBP before 90 min, but finally leveled off after 90 min and a 27.0% reduction of pepsin digestibility was observed at 120 min (Fig 4A). On the other hand, the Bradford protein assay also revealed a delayed digestibility by 8.0% at the initial phase at 60 min (Fig 4B), implicating the cleavage site on the SBP to produce larger peptide fragments had been completely blocked by *DAB* due to the formation of the [*DAB*×*SBP*_gross_]_conjugate_ conjugate. Results have clearly implied a solid evidence supporting strongly our hypothesis that the adduct [*DAB*•*SBP*]_*adduct*_ and the *DAB*-*SBP* adsorption conjugate, [*DAB*×*SBP*_gross_]_conjugate_, tend to enhance the injury to the gastroduodenal system.

As already mentioned, the digestibility of dye-protein adduct can be reduced depending on the adduct characteristics [14].

We suggest firstly that *DAB* being a cationic dyestuff forms ion-pairs between the cationic dyestuff and an negatively–charged surface ions on biomacromolecules like SBP resulting in formation of an electroactive liquid membrane which retards the transport of ions and signals. Similar theoretical basis has been described for ion selective electrode fabrication [29]. Moreover, the surface of pepsin is more negatively charged [30] which would facilitate the adduct formation. Secondly, a coordination complex could occur between DAB and pepsin active site. We suggest that the well oriented [*DAB*•*SBP*]_*adduct*_ adduct would fit better on the active site of pepsin (Fig 4C) than the *DAB*-*SBP* conjugates that are usually formed by random conjugation. Saeed et al.[14] reported the digestibility of dye-protein adduct can be reduced depending on the adduct characteristics [14].

### Correlation of the atypical adsorption isotherm with the toxicity

The experimentally acquired physicochemical parameters for each phase are listed in Table 3. (As for the mathematical deduction, please be referred to Section A1 in S1 Appendix).

**Table 3:** Assignment of the three adsorption isotherms and its related parameters.

Phase	Phase A	Phase B	Phase C
Stoichiometrics^a^, mg/L	*DAB*: 0.2253; *SBP*: 0–40	*DAB*: 0.2253; *SBP*: 40–117	*DAB*: 0.2253 *SBP*: 117–220
State description	homogenous colloidal adduct	a transition state	solid adsorption conjugate
Adsorption coefficient	*k*_*fA*_ = ∞	Non-determinate	*k*_*aC*_ = 3.420×10^−3^
Desorption coefficient	*k*_*rA*_ ≈ 0	Non-determinate	*k*_*dC*_ = 0.106 mg/mL
Equilibrium constant	*K*_*eqA*_ = ∞	Non-determinate	*K*_*eqC*_ = 3.23×10^−2^ mL/mg

^a^*DAB*: methyl yellow. *SBP*: soybean protein.

The adsorption coefficients in Phase A, B, and C (S1 Fig) were *k*_*fA*_ = ∝, non-determinate, and *K*_*aC*_ = 3.42×10^−3^; the desorption coefficients were *k*_*rA*_ 2248 0, non-determinate, and *k*_*dC*_ = 0.106 mg/mL; and the equilibrium constants were *K*_*eqA*_ = ∝, non-determinate, and *K*_*eqC*_ = 3.23×10^−2^ mL/mg, respectively (Table 3).

As already mentioned, cationic dyes forms dye–protein adducts in biological fluids. Its primary *in vivo* toxicity arises from adduct or complex type associated with protein targets [13,14], while the digestibility of dye-protein adduct can be reduced depending on the adduct characteristics [14].

## Conclusions

The toxicity of *DAB* can be enhanced when forming adduct with the soybean protein (*SBP*) (i.e. *DAB-SBP*). The adsorption behavior of DAB onto SBP reveals a three phase adsorption isotherm. In phase A, the soluble hydrocolloid [*DAB-SBP*] produced by 1:1 molar ratio is suggested to be the most toxic as evidenced by the *in vitro* pepsin digestibility test and the *in vivo* animal experiment with Sprague-Dawley rats. The main organs affected by [*DAB-SBP*] are duodenum, liver, kidneys, and mitochondria. The typical Langmuir adsorption can only be perceived in Phase C (SBP ≥117mg/mL). The equilibrium constant of Phase A is infinitely large, implicating formation of extremely stable [*DAB-SBP*] adduct, while Phase C reveals to be only moderately strong adsorption. To our knowledge, this is the first report demonstrating the augmented Gastro-Duodenal-Hepatic toxicity of DAB when forming adduct with soybean protein.

## Supporting information

S1 Appendix Correlation of the atypical adsorption isotherm with the toxicity. (DOCX)

S1 Fig Correlation of differentiated toxicity with the physical chemical behavior of methyl yellow adsorption on soybean protein, and b) The quantified adsorption isotherm of Phase C. a) Whole range adsorption isotherm. b) The characteristic adsorption isotherm of Phase C.
Phase A: the DAB-colloidal adduct phase, The adduct [*DAB*•*SBP*]_*adduct*_ exhibits transparent optical density as a whole, no solid precipitate formed after centrifuged at 14000×g for 6h. Phase B: the transition region between Phase A to Phase C. Phase C: The adsorption isotherm of gross *SBP* revealed a conventional solid adsorption isotherm.
(TIF)
