# Correction: Aminoazo dye-protein-adduct enhances inhibitory effect on digestibility and damages to Gastro-Duodenal-Hepatic axis

**DOI:** 10.1371/journal.pone.0196026

**Published:** 2018-04-12

**Authors:** Li-Yun Lin, Chiung-Chi Peng, Yeh Chen, Boa-Chan Huang, Chun Chao Chang, Robert Y. Peng

There is an error in reference 13. The correct reference is: Tacal Ö, Özer I (2004). Adduct-forming tendencies of cationic triarylmethane dyes with proteins: metabolic and toxicological implications. J Biochem Mol Toxicol 18:253–256.
