# Supplementary material for: Aminoazo dye-protein-adduct enhances inhibitory effect on digestibility and damages to Gastro-Duodenal-Hepatic axis
Source: PLoS One. 2017 Apr 21;12(4):e0170555. doi: 10.1371/journal.pone.0170555 (PMC5400237; doi:10.1371/journal.pone.0170555)
Supplement: S1 Appendix — (DOCX) [file pone.0170555.s001.docx]

**S1 Appedix**

**Section A1. Correlation of the atypical adsorption isotherm with the toxicity**

S1 Fig (in Supporting Information) shows atypical adsorption isotherm of *DAB* on *SBP* at 25^o^C, which amazingly comprised of three different adsorption phases (S1 Fig). Phase A occurred at the soybean protein (*SBP*) concentrations below 40 mg/mL (S1a Fig). In Phase A, no any sediment was obtainable even centrifuged at 14000×g for 6 h, hence this Phase A was also denoted as ‘The Colloidal Adduct Phase’. Phase B was denoted as the transition phase, in which the adsorption isotherm appeared in a manner as the intermediate between Phase A and Phase C. The Phase B occurred at *SBP* concentration within 40 mg/mL to 117 mg/mL. Phase C covered a range from 117 mg/mL up to 220 mg/mL, characteristically exhibiting a typical conventional adsorption isotherm ( S1 Fig).

The rapid surface binding that is mainly electrostatic in nature (like Phase A) is followed by a slower second phase in which *DAB* finds a second site (like Phase C). The interaction at this stage is dominated by the ‘hydrophobic bonding’ as described by Dunn[30]

**A1-1 *Phase A, formation of* [*DAB*•*SBP*]_adduct_ *hydrocolloids***

Below 40 mg/mL of *SBP*, the optical density of the mixture *DAB* and *SBP* increased with the amount of *SBP* present and exhibited a sharp linearity in Phase A (S1a Fig), implicating clearly that a new chemical species with a close stoichiometric relationship between *DAB* and *SBP* could have been produced. This colloidal adduct characteristically could not be precipitated even by centrifugation at 14000×g for 6 h. Judging from its unique physicochemical behaviors like the light absorbance and the sedimentation pattern, it was considered to be due to the formation of the adduct, [*DAB*•*SBP*]*_adduct_* (Phase A, S1 Fig).

In Phase A, the formation of [*DAB*•*SBP*]*_adduct_* follows equation (1).

………………………………..……..(1)

Where is the formation rate constant of adduct [*DAB*•*SBP*]*_adduct_*, and *k_rA_* is the rate constant of [*DAB*•*SBP*]*_adduct_* of reverse reaction, and

……………………………….….(2)

Similarly for the reverse reaction,

……………………..…………………..(3)

At equilibrium, the relationship exists in Phase A:

………………………….…….....(4)

Whereis the equilibrium constant of Phase A. Alternatively, we have the formation of adduct as

……………………………….…..…..(5)

Literature has reported that some of the dyes-protein adducts characteristically showed a colorless adduct (i.e. lower optical density than the blank) as observed in the malachite green (MG^+^)-protein adducts [9]. Contrary to this, we showed the optical density of [*DAB*•*SBP*]_adduct_ was substantially higher than the reference (i.e. the OD of the adduct has stronger absorbance than the blank *DAB*) (Curve 1 in Phase A of S1a Fig), underlying the creation of novel strong absorbing chromophore(s) between the *DAB* and the *SBP* molecules. At a given *DAB* concentration, the dose dependent linearity of slope indicated that the free *SBP* was nearly consumed up and being absent in Phase A, hence the equilibrium constant *K_eqA_* can be considered infinitely large (equation 2), i.e. *k_fA_*→∝, or *K_eqA_* maintained at ≈ ∞ until the *SBP* concentration reached up to 40 mg/mL (S1 Fig). In other words, below a concentration of *SBP* 40 mg/mL the stoichiometric adsorption of *DAB* onto *SBP* in reality can be considered completely furnished and the only existing chemical species in the system would only be [*DAB*•*SBP*]_adduct_ (equation 4). Tacal and Özer[13] previously had implicated similar phenomenon occurring between the biological proteins and the electrophilic cationic triarylmethane dyes [13].

Now the problem arises ‘What is the stoichiometry for the formation of such an adduct?’ Assuming the stoichiometric factor to be 1 with the reason that it presented a perfect linearity (slope 1 in Phase A, S1 Fig), equation (6) was recommended to calculate the mean molecular weight of *SBP*.

……………………………………(6)

Where *MW_SBP_* is the molecular weight of *SBP*. *N_Av_* is the Avogadro’s number which is 6.022×10^23^mol^-1^. *W_DAB_* is the weight of *DAB* used in this adsorption experiment, which is 1.1265×10^-4^g. *M_DAB_* is the molecular weight of *DAB*, which is 225.32. The value 0.2 g is the maximum amount of *SBP* behaving as ‘a true colloidal adduct’ in Phase A.

In this system, the amount of $DAB$(MW = 225.32) used was 1.1265 ×10^-4^g, which was equivalent to 5.0×10^-7^ mol. Multiplication of this figure with the Avogadro’s number (*N_Av_* =6.022×10^23^ molecules/mol) yielded 3.011×10^17^ molecules. As the stoichiometric titration curve revealed a linearity with a straight slope (slope 1 of S1 Fig), the stoichiometric factor seemingly could be assumed to be 1, which implied that the number of molecules of$SBP$present in the same system (i.e. 200 mg = 0.200g) was also 3.011×10^17^ molecules. From these data the ‘mean molecular weight’ of SBP was obtained to be [0.200g/(3.011×10^17^ molecules)]×6.022×10^23^ molecules/mol = 4.0×10^5^ g/mol. Monagle et al.[31] describes soy protein aggregates that have a substantial portion of their proteins exhibit mean molecular weight ranging from 1,000 to 380,000 [31], a result strongly support the 1:1 molar stoichiometry in the adduct [*DAB*•*SBP*]_adduct_ as initially proposed in the above mentioned.

**A1-2 *Phase B - a transition state***

The adsorption isotherm found in Phase B behaved a transition type (S1 Fig) which apparently presented two adsorption types, i.e. a) the adsorption isotherm as shown in Eq. 1, and b) the adsorption of free *DAB* on to the gross *SBP_gross_*. Thus in Phase B there could co-exist several different chemical species, like small amount of free gross *SBP_gross_*, the constant amount of [*DAB*•*SBP*]_adduct_, and the adsorbed *DAB* on the existing small amount of gross *SBP_gross_*, and the adsorbed conjugate [*DAB*×*SBP*_gross_]_conjugate_.

**A1-3 *Phase C, formation of*** [***DAB*×*SBP***_gross_]_conjugate_

In Phase C, the adsorption behavior occurred as conventional as shown in equation (7)

……………………………...(7)

The macroscopic adsorption capability of$SBP$could be calculated from the slope curve 2 (S1b Fig)

………………………….………..(8)

As generally defined, the adsorption of protein *SBP* in Phase C implies that the rate of adsorption is proportional to (1-*θ*)[*X*], where [*X*] is the concentration of *SBP* present in the system, and *θ* is the surface area fraction already occupied by *DAB* molecules, and only the remaining surface area fraction (1-*θ*) is available for *DAB* adsorption, thus

…………………………………………….……….…..(9)

One of the boundary condition is and$\theta=0$at $X=117mg/mL$(curve 2 of Phase C, S1b Fig), substitution of these figures into Eq. (9) yields the proportional coefficient of the forward adsorption in phase C,

…………………………………………………………...(10)

On the other hand, at equilibrium (curve 3 in Phase C, S1b Fig), the rate of adsorption and the rate of desorption should be equal, i.e.

………………….(11)

On rearrangement yields

………………..(12)

Substitution of the boundary parameters (S1b Fig) at equilibrium into Eq. (12) gives

…….…..(13)

And the equilibrium constant is

……………………………………….…….(14)

As protein molecules are flexible when dissolved or suspended, its conformation constantly changes and the effective fraction of bare surface (1-*θ*) will be also changing as well during the experiment, but the quantity of the bare surface (1-*θ*) will not change at a given concentration of *SBP* and temperature, i.e.

$(1-\theta)=f[X]$…………………………………………………………..…(15)

Thus the amount of *DAB* adsorbed onto *SBP* (*X*) throughout the whole course in reality should obey the relationship

………………………………………….……..(16)

or

…………………………………………………...(17)

Where *R_Xa_* is the amount of *DAB* adsorbed onto the *SBP* surface. *k_ac_*, as in the above mentioned, is the proportional coefficient of the forward adsorption in phase C.

On rearrangement and integration, and substituting the boundary conditions shown in S1b Fig into equation (17) leads to

………………………………..….…….(18)

Alternately, the amount of *DAB* desorbed (*R_Xd_*) from *SBP*(*X*) is proportional only to the number of molecules already adsorbed onto the surface fraction *θ*, which in turn actually is proportional to the fraction of surface covered:

……………………………………………..………(19)

Rearrangement and integration of equation (19) leads to

…………………………………………….(20)

At equilibrium, equation (18) = equation (20), which leads to

…………………………………….…(21)

or

……………………………………………(22)

Rearrangement of equation (22) gives

…………………………………..…...(23)

Substitution with 3.226×10^-2^ mg/mL forleads to

………………………………….….……(24)

To summarize, the adsorption behavior of *DAB* to occupy a fraction of surface *θ* on the *SBP*(*X*) is a function of the adsorption-desorption equilibrium constant *K_eqC_*, and the ratio of the amount adsorbed (*R_Xa_*) to the amount desorbed (*R_Xd_*). Equations (23) and equation (24) in some respects are similar to the typical Langmuir isotherm.
