# Supplementary figures and images for: Aminoazo dye-protein-adduct enhances inhibitory effect on digestibility and damages to Gastro-Duodenal-Hepatic axis
Source: PLoS One. 2017 Apr 21;12(4):e0170555. doi: 10.1371/journal.pone.0170555 (PMC5400237; doi:10.1371/journal.pone.0170555)

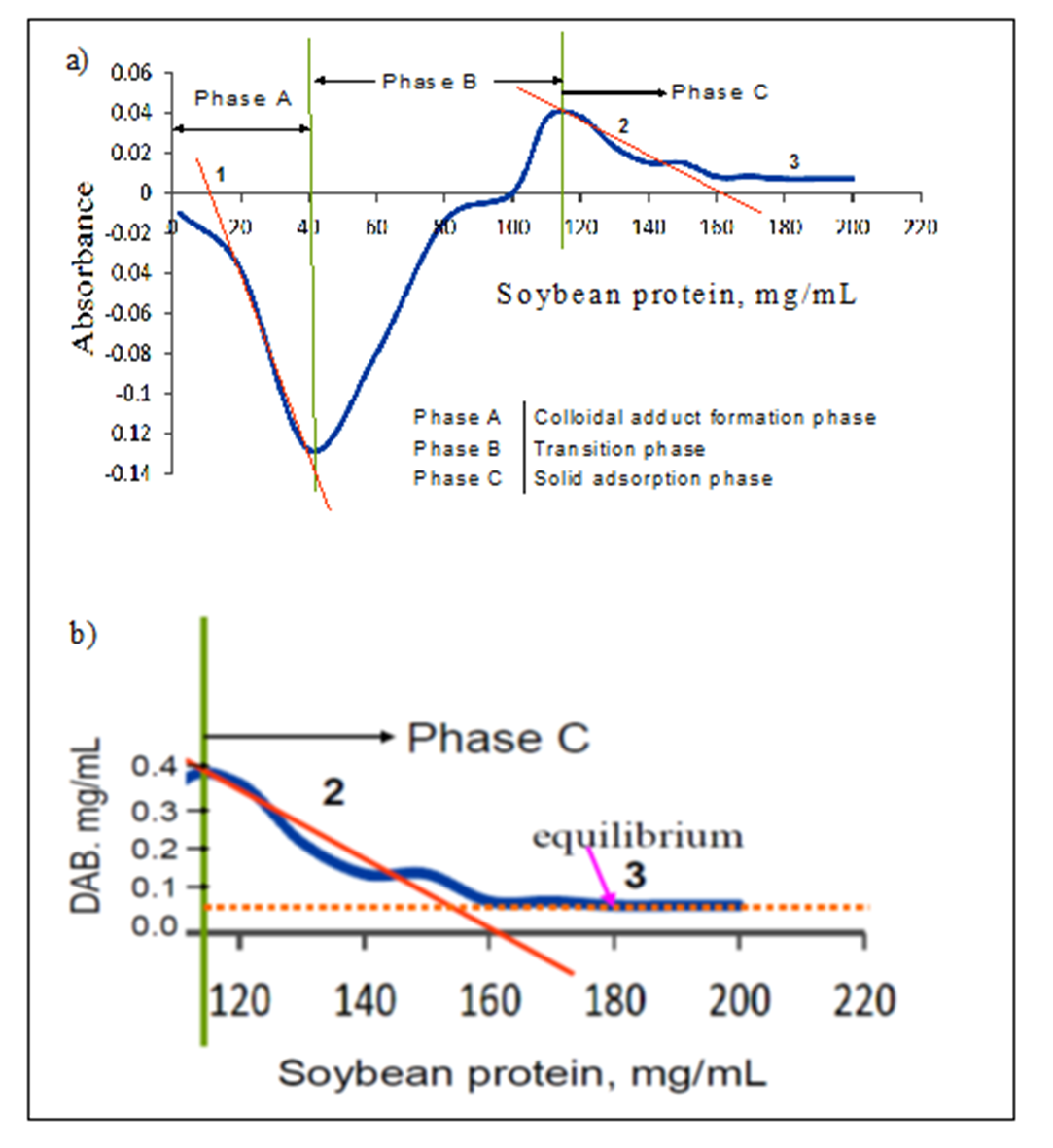

Supplement: S1 Fig — a) Whole range adsorption isotherm. b) The characteristic adsorption isotherm of Phase C. Phase A: the DAB-colloidal adduct phase, The adduct [DAB•SBP]adduct exhibits transparent optical density as a whole, no solid precipitate formed after centrifuged at 14000×g for 6h. Phase B: the transition region between Phase A to Phase C. Phase C: The adsorption isotherm of gross SBP revealed a conventional solid adsorption isotherm. (TIF) [file pone.0170555.s002.tif]
